# Easily-controllable, helper phage-free single-stranded phagemid production system

**DOI:** 10.1186/s41021-022-00254-1

**Published:** 2022-11-16

**Authors:** Tetsuya Suzuki, Hiroyuki Kamiya

**Affiliations:** Graduate School of Biomedical and Health Sciences, Hiroshima University, 1-2-3 Kasumi, Minami-ku, Hiroshima, 734-8553 Japan

**Keywords:** Single-stranded DNA, Phagemid, Helper phage, Anion-exchange column

## Abstract

**Background:**

Single-stranded (ss) DNAs are utilized in various molecular biological and biotechnological applications including the construction of double-stranded DNAs with a DNA lesion, and are commonly prepared by using chimeric phage-plasmids (phagemids) plus M13-derived helper phages. However, the yields of ss DNA with these methods are poorly reproducible, and multiple factors must be optimized.

**Results:**

In this report, we describe a new arabinose-inducible ss phagemid production method without helper phage infection. The newly exploited DNA derived from VCSM13 expresses the pII protein, which initiates ss DNA synthesis, under the control of the *araBAD* promoter. In addition, the packaging signal is deleted in the DNA to reduce the contamination of the phage-derived ss DNA. The phagemid DNA of interest, carrying the M13 origin of replication and the packaging signal, was introduced into bacterial cells maintaining the modified VCSM13 DNA as a plasmid, and the ss phagemid DNA production was induced by arabinose. The DNA recovered from the phage particles had less contamination from VCSM13 DNA, as compared to the conventional method. Moreover, we extended the method to purify the ss DNAs by using an anion-exchange column, to avoid the use of hazardous chemicals.

**Conclusion:**

Using this combination of methods, large quantities of phagemid ss DNAs of interest can be consistently obtained.

## Background

Single-stranded (ss) DNAs are the optimal templates for various DNA polymerase-based molecular biological and biotechnological applications, such as DNA sequencing and site-directed mutagenesis [1–4]. Among such applications, the construction of double-stranded (ds) DNAs with a DNA lesion, from ss circular phage/phagemid DNAs, is quite useful to study the effects of DNA modifications on DNA transactions, including mutagenesis, repair, replication, and transcription [5–8]. We have studied the molecular mechanisms of mutagenesis, DNA repair, and translesion DNA synthesis by using shuttle phagemids with a DNA lesion at a pre-determined position, and successfully clarified some mutagenesis processes of damaged guanine bases and an abasic site, at both the lesion sites and positions distant from the lesions [9–16]. The latter untargeted mutations can only be identified when the lesions are located at predefined positions. Moreover, ss linear DNAs are used as donor DNAs for genome editing, with and without artificial nucleases and hybridization probes, to detect specific complementary DNA sequences [17–26]. We have prepared long ss linear DNA fragments of interest from ss circular phagemid/phage DNAs by hybridizing scaffold oligonucleotides to generate ds regions in targeted restriction sites, followed by restriction enzyme digestion [18, 25, 26].

To prepare ss phagemid DNAs, many researchers including us have utilized methods in which an F′ *Escherichia coli* strain with a phagemid containing the M13 or f1 origin of replication (*ori*) is infected with a helper phage [27, 28]. However, the yields of ss DNA are poorly reproducible, and various conditions, such as titers of helper phages, multiplicities of infection (MOI), and densities of *E. coli* at the time of infection, must be controlled to consistently obtain sufficient quantities of ss phagemids in the conventional method [29]. In addition, the ss DNA production may be lower, and contaminated with the helper phage-derived ss DNAs, depending on the constitutions of phagemids. Moreover, hazardous chemicals, phenol and chloroform, are commonly used to extract ss DNAs from produced phages, and their carry-over could influence the downstream applications.

We exploited a new VCSM13-based DNA to solve these problems. The M13 pII protein is required to initiate rolling circle replication. We replaced the promoter driving the *pII* gene with the *araBAD* promoter, which intrinsically regulates the arabinose operon [30]. We also eliminated the packaging signal in the M13 intergenic sequence to reduce the contamination by the phage-derived ss DNA [31, 32]. We used *E. coli* cells bearing this DNA as a plasmid. When phagemid DNA of interest is introduced into this *E. coli* host, it only produces phages containing the ss phagemid in the presence of arabinose, which induces the expression of the pII protein. In addition, an anion-exchange column was used to purify the ss DNA extracted from phages, without phenol and chloroform. By combining these methods, we consistently prepared large quantities of desired ss phagemid DNAs, without considering detailed conditions and using hazardous chemicals, even when the yield of the ss phagemid of interest is low by the conventional method.

## Materials and methods

### Materials

The oligodeoxynucleotides used as PCR primers were purchased from Integrated DNA Technologies (Coralville, IA, USA), and Hokkaido System Science (Sapporo, Japan) in purified forms. VCSM13 was obtained from Agilent Technologies (Santa Clara, CA, USA). The 1 kbp ladder DNA size marker was from GeneDireX (Taipei, Taiwan).

### Construction of M13-based DNA for arabinose-inducible phage production

The sequence from the ribosome binding site to the *Hin*cII site of the M13 phage *pII* gene was amplified by PCR, using VCSM13 as the template and the primers pII RBS Fw (5′-TTTGGATCAACCGGGGTACATATGA) and pII(HincII) Rv (5′-ATAGTAGTAGCGTTAACATCC). The *araBAD* promoter sequence with the *araC* gene was amplified by PCR, using *E. coli* BL21(DE3) genomic DNA as the template and the primers pBAD Fw (5′-CCCCCCCCCCCTGCATGCATAATGTGCCTGTCAAA) and Rv (5′-CCCGGTTGATCCAAAAAAACGGGTATGGAG). These two PCR fragments were assembled with *Pst*I- and *Hin*cII-digested VCSM13 by using the GeneArt Seamless Cloning and Assembly Enzyme Mix (Thermo Fisher Scientific, Waltham, MA, USA), and the resultant plasmid was named VCSM13(P_BAD_-pII).

Two DNA fragments around the M13 intergenic sequence were amplified by PCR, using VCSM13 as the template and the primer sets M13dPS Fw (5′-AAGCGAATT CGCAGCGTGACCGCTACACT) plus M13IGNaeI Rv (5′-TTGACGGGGAAAGCCGGCGA) and pIV-PsiI Fw (5′-TGGCCTCACTGATTATAAAA) plus M13dPS Rv (5′-GCTGCGAATTCGCTTAATGCGCCGCTACAG), and then the two fragments were combined by overlap extension PCR. The DNA fragment digested by *Psi*I and *Nae*I was ligated with VCSM13(P_BAD_-pII) digested by the same enzymes to remove the packaging signal in the M13 intergenic sequence, and the resultant plasmid DNA was named VCSM13ΔPS(P_BAD_-pII). *E. coli* HB101 was transformed by VCSM13ΔPS(P_BAD_-pII).

### Phage production by arabinose-induction

*E. coli* HB101 bearing VCSM13ΔPS(P_BAD_-pII) was transformed with 1 ng of pBS189R-BsmBI or pSB189L-BsmBI (containing the M13 intergenic sequence) [11], and plated onto LB agar plates containing 100 μg/mL ampicillin, 25 μg/mL kanamycin, and 0.2% glucose. A single colony was inoculated into 2 mL of LB containing 100 μg/mL ampicillin, 25 μg/mL kanamycin, and 0.2% glucose, and cultured overnight. The overnight culture was diluted 50-fold in 2 × YT medium containing 100 μg/mL ampicillin and 25 μg/mL kanamycin, and cultured at 37 °C with shaking until the optical density at 610 nm (OD_610_) reached the targeted values. Arabinose was added to the culture at final concentrations of 0.002, 0.02%, or 0.2%, and the culture was incubated further at 37 °C overnight with shaking. For middle-scale phagemid isolation, a 50 mL culture in a 200 mL baffled flask was used. For small-scale phagemid isolation, a 7 mL culture in an 18 × 180 mm test tube was used.

### Phage production by VCSM13 infection (conventional method)

A single colony of *E. coli* JM109/F′ bearing pBS189KR-BsmBI or pSB189KL-BsmBI was inoculated into 2 mL of LB containing 100 μg/mL ampicillin and cultured overnight. The overnight culture was diluted 1000-fold in 2 × YT medium containing 100 μg/mL ampicillin, and cultured at 37 °C with shaking until it reached the targeted OD_610_ value. VCSM13 helper phage was added at a multiplicity of infection (MOI) of 20:1, and the culture was continued at 37 °C for 2 h with shaking. After phage infection, kanamycin was added at a 25 μg/mL final concentration, and the culture was incubated further at 37 °C overnight with shaking.

### Determination of single-stranded DNA yield

The phage particles precipitated by 20% polyethylene glycol (PEG)-6000/2.5 M NaCl from 1 mL of each culture were resuspended in 50 μL of TE (pH 8.0) containing 1 μL of ≥600 mAnson units/mL proteinase K (TaKaRa, Kusatsu, Japan) and 0.1% SDS, and then incubated at 42 °C for 1 h. One microliter of each sample was electrophoresed on a 1% agarose TAE gel, and the DNA was stained by GelRed Nucleic Acid Gel Stain (Biotium Inc., Fremont, CA, USA).

### Middle-scale single-stranded DNA purification on an anion-exchange column

The phage particles produced from a 50 mL culture were precipitated by adding a one-tenth volume of 20% PEG-6000/2.5 M NaCl solution to the supernatant of the overnight culture. The solution containing the phage precipitate was centrifuged at 12,000 rpm for 15 min at 4 °C to concentrate the phage. The pellet was resuspended in 1 mL of 10 mM Tris-HCl (pH 8.0), and then 10 μL of 300 mM MgCl_2_, 5 units of recombinant DNase I (TaKaRa) and 10 mg of RNase A (Nacalai Tesque, Kyoto, Japan) were added, followed by an incubation at room temperature for 1 h to remove contaminations of bacterial nucleic acids in the supernatant. Afterwards, 6.6 μL of 0.5 M EDTA (pH 8.0) was added to the suspension, and then 5 μL of ≥600 mAnson units/mL proteinase K and 50 μL of 10% SDS were added, and the mixture was incubated at 50 °C for 1 h. After this incubation, 0.5 mL of Buffer P3 (QIAGEN, Venlo, Netherlands) was added, and the solution was then centrifuged at 12,000 rpm for 5 min at 4 °C. The supernatant was applied to a QIAGEN-tip20 column pre-equilibrated with Buffer QBT, and the column was washed with 2 mL of Buffer QC twice. The ss DNA was eluted with 1.6 mL of Buffer QF (QIAGEN) pre-warmed to 50 °C, and then precipitated and resuspended in an appropriate volume of solution.

## Results

### Arabinose-induced phage production conditions

**Fig. 1:**
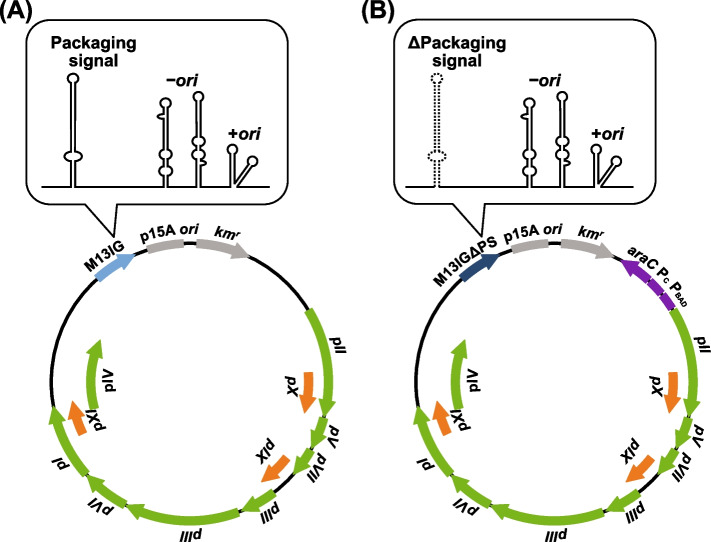
The (**A**) VCSM13 helper phage DNA and (**B**) VCSM13ΔPS(P_BAD_-pII) DNA used in this study. The secondary structure of the M13 intergenic sequence is indicated in balloons. Dashed line indicates deletion. *Ori*, replication origin; *km*^*r*^, kanamycin resistance gene; IG, intergenic sequence; P_BAD_, *araBAD* promoter; P_C_, *araC* promoter

**Fig. 2:**
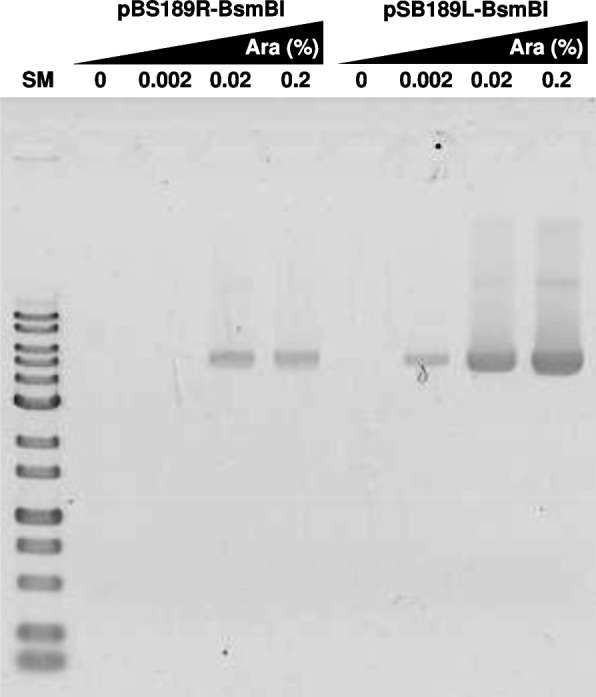
GelRed-stained 1% agarose gel image of ss DNAs produced by arabinose induction. The phages were produced by arabinose at indicated concentrations when the OD_610_ values of *E. coli* cultures (HB101/VCSM13ΔPS(P_BAD_-pII) bearing pBS189R-BsmBI or pSB189L-BsmBI) reached between 0.4 and 0.5. The reverse image is shown. Ara, arabinose; SM, 1 kbp ladder DNA size marker

**Fig. 3:**
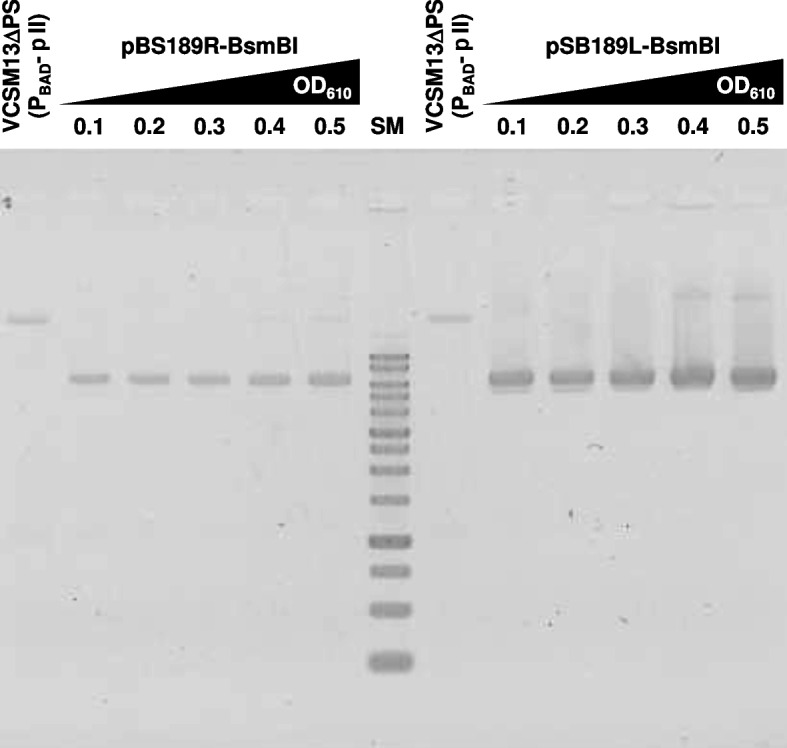
GelRed-stained 1% agarose gel image of ss DNAs produced by arabinose induction. The phages were produced by 0.2% arabinose addition at the indicated OD_610_ values of the *E. coli* cultures (HB101/VCSM13ΔPS(P_BAD_-pII) bearing pBS189R-BsmBI or pSB189L-BsmBI). The reverse image is shown. The “VCSM13ΔPS(P_BAD_-pII)” lanes indicate the ss DNA of the plasmid for contamination check. SM, 1 kbp ladder DNA size marker

We developed the new VCSM13-derived DNA, expressing the pII protein under the control of the *araBAD* promoter and lacking the packaging signal (Fig. 1). The VCSM13ΔPS(P_BAD_-pII) DNA is maintained as a plasmid in *E. coli* HB101, and phagemid DNA of interest was introduced into *E. coli* HB101/VCSM13ΔPS(P_BAD_-pII). Various culture conditions can affect phage production. In order to determine the optimal conditions for phage production, we examined the effects of arabinose concentration and *E. coli* culture density on the ss DNA yields of two phagemids, pBS189R-BsmBI and pSB189L-BsmBI (Figs. 2 and 3). These phagemids were used to compare the new method and the conventional method, since the ss pSB189L-BsmBI is reproducibly and efficiently obtained by VCSM13 helper phage infection, but the ss pBS189R-BsmBI is not, as described below. The addition of arabinose to the culture induces the pII protein expression, resulting in the production of phage containing the ss phagemid of interest. Neither pBS189R-BsmBI nor pSB189L-BsmBI ss DNA was produced without arabinose. The ss forms of pBS189R-BsmBI and pSB189L-BsmBI were observed in cultures containing 0.02 and 0.002% arabinose, respectively. The quantities of ss pBS189R-BsmBI were similar at 0.02 and 0.2% arabinose. In contrast, the quantities of ss pSB189L-BsmBI dose-dependently increased up to 0.2% arabinose. Obviously, the yields of ss pSB189L-BsmBI were higher than those of ss pBS189R-BsmBI at all arabinose concentrations. We used 0.2% arabinose in the experiments described below.

Next, we examined the effects of *E. coli* densities on the ss DNA yields (Fig. 3). The OD_610_ values of the culture were varied between 0.1 (early logarithmic phase) and 0.5 (middle logarithmic phase). The yields of ss DNA increased in a density-dependent manner for both phagemids. However, the ss DNAs were moderately produced even at low cell densities. Again, the yields of ss pSB189L-BsmBI were higher than those of ss pBS189R-BsmBI at all culture densities examined. We decided to initiate arabinose induction at an OD_610_ value of 0.4–0.5.

### Comparison of arabinose-induced and conventional helper phage infection methods

**Fig. 4:**
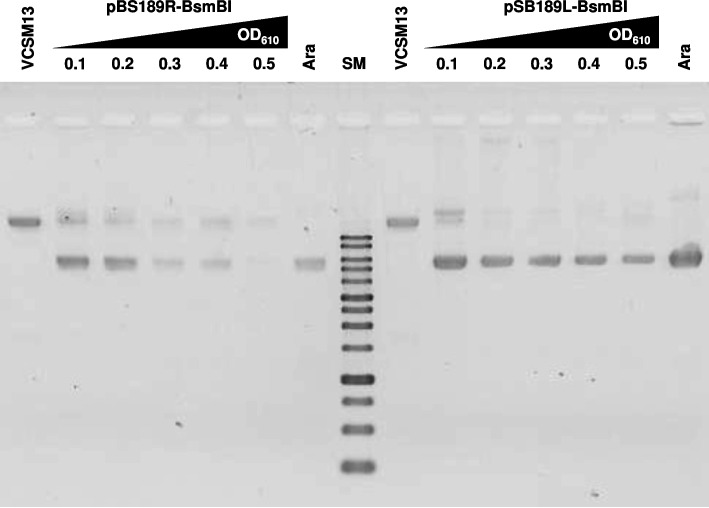
GelRed-stained 1% agarose gel image of ss DNAs produced by VCSM13 infection. The phages were produced by VCSM13 infection at the indicated OD_610_ values of the *E. coli* cultures (JM109 bearing pBS189R-BsmBI or pSB189L-BsmBI). The reverse image is shown. The “VCSM13” lanes indicate the ss DNA of the helper phage. The “Ara” lanes indicate ss DNAs of the indicated phagemids produced by arabinose induction (0.2% arabinose at OD_610_ = 0.4). SM, 1 kbp ladder DNA size marker

We compared the arabinose-induction method with the conventional helper phage infection method. The frequently used VCSM13 helper phage was used to infect *E. coli*/F′ (JM109) bearing pBS189R-BsmBI and pSB189L-BsmBI at OD_610_ values ranging from 0.1 to 0.5 (Fig. 4). In contrast to the new method, the production of both ss phagemids was less when the higher culture densities were infected with VCSM13. In addition, the yield variations according to the culture density were larger than those observed with arabinose induction. Importantly, smaller amounts of the extra DNAs, including the helper phage ss DNA, were produced in the arabinose induction method, as compared to the VCSM13 infection. These results indicated that ss phagemids were more efficiently produced, with less contamination, at a wide range of culture densities by the arabinose induction method than by the conventional helper phage infection method.

### Purification of single-stranded phagemid vector on an anion-exchange column

**Fig. 5:**
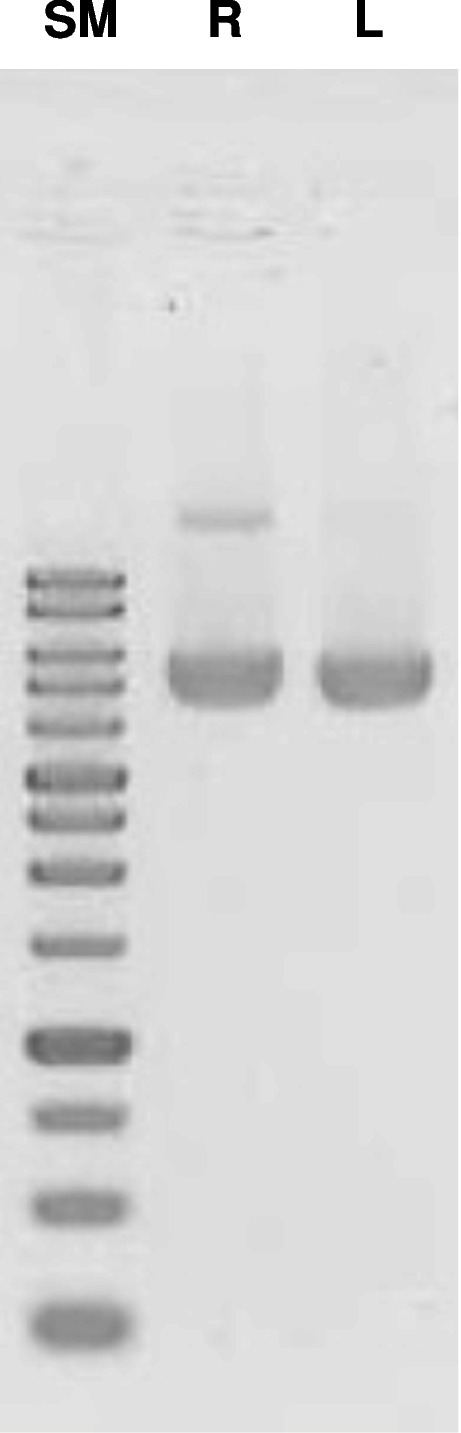
GelRed-stained 1% agarose gel image of ss DNAs purified by anion-exchange chromatography on a middle scale. The phages were produced by 0.2% arabinose when the OD_610_ values of *E. coli* cultures (HB101/VCSM13ΔPS(P_BAD_-pII) bearing pBS189R-BsmBI or pSB189L-BsmBI) reached between 0.4 and 0.5. The reverse image is shown. R, pBS189KR-BsmBI; L, pSB189KL-BsmBI; SM, 1 kbp ladder DNA size marker

**Fig. 6:**
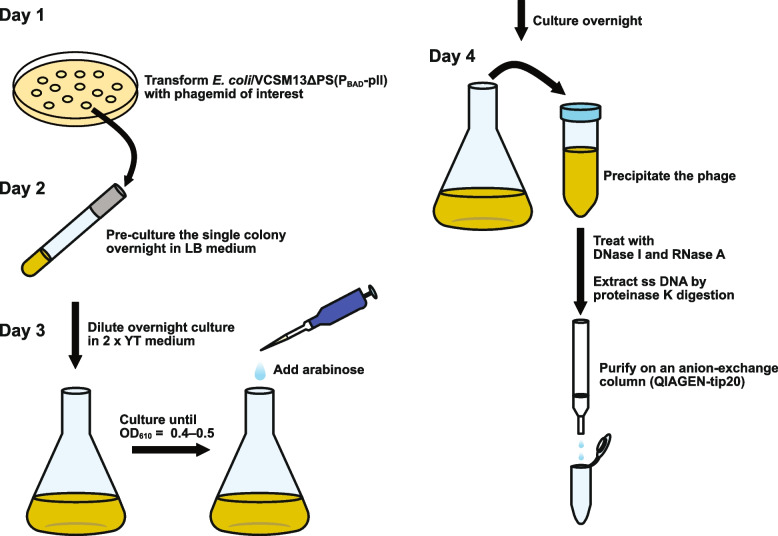
Schematic diagram of the experimental procedures for the production of phages containing the ss phagemid of interest and the DNA purification on an anion-exchange column. Day 1, Transformation of *E. coli* bearing VCSM13ΔPS(P_BAD_-pII) with phagemid of interest. Day 2, Pre-culture of a single colony in LB medium containing optimal antibiotics and glucose. Day 3, Culture in 2 × YT medium and induce phage production by arabinose. Day 4, Precipitation of the phage and purification of the ss phagemid on an anion-exchange column

We attempted to purify ss DNAs by using an anion-exchange column (QIAGEN-tip20) on the middle scale, without hazardous materials (Fig. 5). A schematic diagram of the experimental procedure is shown in Fig. 6. The phage particles resuspended after PEG-precipitation were treated with DNase I and RNase A to remove the contaminating nucleic acids in the culture supernatant, before phage lysis. The yields of ss pBS189R-BsmBI and ss pSB189L-BsmBI were 0.7 (± 0.1) and 2.1 (± 0.3) μg/mL culture, respectively. Double-stranded DNAs bearing a modified base at a pre-determined position were successfully constructed by using ss DNAs purified with this system [33].

## Discussion

We developed a new ss phagemid production method using arabinose-mediated pII protein expression, instead of helper phage infection. Since the ss DNA yields were constant and independent of the *E. coli* culture density at the time of arabinose addition, experiments to define the optimal conditions are unnecessary. In addition, considerations of some factors that influence the ss DNA yields in the helper phage methods, such as phage titer, MOI, and loss of F′ episome, are not required. Indeed, by using the newly developed method we obtained reasonable quantities of ss pBS189R-BsmBI DNA, which is difficult to produce by the conventional method.

Phenol and chloroform are commonly used to extract ss DNA from PEG-precipitated phage. The purification procedure using an anion-exchange column, instead of these hazardous chemicals, allows us to safely recover highly purified ss DNAs. We obtained approximately 1 μg of ss DNA per mL culture by the combination of these growth and purification systems. Thus, abundant quantities of ss phagemids can be obtained from a middle-scale culture. Moreover, the culture volume can be conveniently scaled up to prepare larger quantities of ss DNAs, since various anion-exchange columns for larger-scale DNA purification are commercially available.

## Conclusions

We have developed a reproducible and safe ss phagemid manufacturing system. This system is a potential tool for various applications requiring large quantities of highly purified ss DNAs.

## Data Availability

The datasets generated and/or analyzed during the current study are available from the corresponding authors on reasonable request.

## Abbreviations

ss: Single-stranded
PEG: Polyethylene glycol

## References

[1] Goszczynski B, McGhee JD (1991). Resolution of sequencing ambiguities: a universal FokI adapter permits Maxam-Gilbert re-sequencing of single-stranded phagemid DNA. Gene.

[2] Trower MK (1996). A protocol for site-directed mutagenesis employing a uracil-containing phagemid template. Methods Mol Biol.

[3] Zoller MJ, Smith M (1983). Oligonucleotide-directed mutagenesis of DNA fragments cloned into M13 vectors. Methods Enzymol.

[4] Zoller MJ, Smith M (1984). Oligonucleotide-directed mutagenesis: a simple method using two oligonucleotide primers and a single-stranded DNA template. DNA.

[5] Bregeon D, Doetsch PW (2006). Assays for transcriptional mutagenesis in active genes. Methods Enzymol.

[6] Bregeon D, Doddridge ZA, You HJ, Weiss B, Doetsch PW (2003). Transcriptional mutagenesis induced by uracil and 8-oxoguanine in Escherichia coli. Mol Cell.

[7] Bregeon D, Peignon PA, Sarasin A (2009). Transcriptional mutagenesis induced by 8-oxoguanine in mammalian cells. PLoS Genet.

[8] Shivji MK, Moggs JG, Kuraoka I, Wood RD (1999). Dual-incision assays for nucleotide excision repair using DNA with a lesion at a specific site. Methods Mol Biol.

[9] Kamiya H, Yamazaki D, Nakamura E, Makino T, Kobayashi M, Matsuoka I (2015). Action-at-a-distance mutagenesis induced by oxidized guanine in Werner syndrome protein-reduced human cells. Chem Res Toxicol.

[10] Suzuki T, Harashima H, Kamiya H (2010). Effects of base excision repair proteins on mutagenesis by 8-oxo-7,8-dihydroguanine (8-hydroxyguanine) paired with cytosine and adenine. DNA Repair (Amst).

[11] Suzuki T, Katayama Y, Komatsu Y, Kamiya H (2019). Large deletions and untargeted substitutions induced by abasic site analog on leading versus lagging strand templates in human cells. Mutagenesis.

[12] Suzuki T, Katayama Y, Komatsu Y, Kamiya H (2021). Similar frequency and signature of untargeted substitutions induced by abasic site analog under reduced human APE1 conditions. J Toxicol Sci.

[13] Suzuki T, Kuramoto Y, Kamiya H (2018). Reduction of Werner Syndrome Protein Enhances G:C --> A:T Transition by O(6)-Methylguanine in Human Cells. Chem Res Toxicol.

[14] Suzuki T, Masuda H, Mori M, Ito R, Kamiya H (2021). Action-at-a-distance mutations at 5′-GpA-3′ sites induced by oxidised guanine in WRN-knockdown cells. Mutagenesis.

[15] Suzuki T, Sassa A, Grúz P, Gupta RC, Johnson F, Adachi N (2021). Error-prone bypass patch by a low-fidelity variant of DNA polymerase zeta in human cells. DNA Repair (Amst).

[16] Suzuki T, Zaima Y, Fujikawa Y, Fukushima R, Kamiya H (2022). Paradoxical role of the major DNA repair protein, OGG1, in action-at-a-distance mutation induction by 8-oxo-7,8-dihydroguanine. DNA Repair (Amst).

[17] Codner GF, Mianne J, Caulder A, Loeffler J, Fell R, King R (2018). Application of long single-stranded DNA donors in genome editing: generation and validation of mouse mutants. BMC Biol.

[18] Kamiya H, Uchiyama M, Piao J, Nakatsu Y, Tsuzuki T, Harashima H (2010). Targeted sequence alteration of a chromosomal locus in mouse liver. Int J Pharm.

[19] Kawai H, Sato K, Shirahama W, Suzuki T, Kamiya H (2020). Single-stranded DNA versus tailed duplex in sequence conversion of lacZalpha DNA. Nucleosides Nucleotides Nucleic Acids.

[20] Lanza DG, Gaspero A, Lorenzo I, Liao L, Zheng P, Wang Y (2018). Comparative analysis of single-stranded DNA donors to generate conditional null mouse alleles. BMC Biol.

[21] Miura H, Gurumurthy CB, Sato T, Sato M, Ohtsuka M (2015). CRISPR/Cas9-based generation of knockdown mice by intronic insertion of artificial microRNA using longer single-stranded DNA. Sci Rep.

[22] Quadros RM, Miura H, Harms DW, Akatsuka H, Sato T, Aida T (2017). Easi-CRISPR: a robust method for one-step generation of mice carrying conditional and insertion alleles using long ssDNA donors and CRISPR ribonucleoproteins. Genome Biol.

[23] Suzuki T, Imada T, Nishigaki N, Kobayashi M, Matsuoka I, Kamiya H (2016). Cleavage of target DNA promotes sequence conversion with a tailed duplex. Biol Pharm Bull.

[24] Suzuki T, Yanai Y, Nishigaki N, Nakatsu Y, Tsuzuki T, Kamiya H (2018). Effects of mismatches distant from the target position on gene correction with a 5′-tailed duplex. J Biosci Bioeng.

[25] Tsuchiya H, Harashima H, Kamiya H (2005). Increased SFHR gene correction efficiency with sense single-stranded DNA. J Gene Med.

[26] Tsuchiya H, Uchiyama M, Hara K, Nakatsu Y, Tsuzuki T, Inoue H (2008). Improved gene correction efficiency with a tailed duplex DNA fragment. Biochemistry.

[27] Swords WE (2003). Preparation of single-stranded DNA from phagemid vectors. Methods Mol Biol.

[28] Trower MK (1996). Preparation of ssDNA from phagemid vectors. Methods Mol Biol.

[29] Petrova L, Gran C, Bjoras M, Doetsch PW (2016). Efficient and reliable production of vectors for the study of the repair, mutagenesis, and phenotypic consequences of defined DNA damage lesions in mammalian cells. PLoS One.

[30] Guzman LM, Belin D, Carson MJ, Beckwith J (1995). Tight regulation, modulation, and high-level expression by vectors containing the arabinose P_BAD_ promoter. J Bacteriol.

[31] Peeters BP, Schoenmakers JG, Konings RN (1987). Comparison of the DNA sequences involved in replication and packaging of the filamentous phages IKe and Ff (M13, fd, and f1). DNA.

[32] Russel M, Model P (1989). Genetic analysis of the filamentous bacteriophage packaging signal and of the proteins that interact with it. J Virol.

[33] Fukushima R, Suzuki T, Komatsu Y, Kamiya H. Biased distribution of action-at-a-distance mutations by 8-oxo-78-dihydroguanine. Mutat Res/Fund Mol Mech. 2022;825:111794 10.1016/j.mrfmmm.2022.111794 36027647

